# The Role of IL-33-Dependent Inflammation in the Tumor Microenvironment

**DOI:** 10.3389/fimmu.2016.00682

**Published:** 2017-01-09

**Authors:** Marie-Hélène Wasmer, Philippe Krebs

**Affiliations:** ^1^Institute of Pathology, University of Bern, Bern, Switzerland; ^2^Graduate School for Cellular and Biomedical Sciences, University of Bern, Bern, Switzerland

**Keywords:** cancer, inflammation, tumor microenvironment, interleukin-33, therapy

## Abstract

There is compelling evidence that inflammation contributes to tumorigenesis. Inflammatory mediators within the tumor microenvironment can either promote an antitumor immune response or support tumor pathogenesis. Therefore, it is critical to determine the relative contribution of tumor-associated inflammatory pathways to cancer development. Interleukin-33 (IL-33) is a member of the IL-1 family of cytokines that is released upon tissue stress or damage to operate as an alarmin. IL-33 has been primarily implicated in the induction of type-2 immune responses. However, recent findings have shown a role of IL-33 in several cancers where it may exert multiple functions. In this review, we will present the current knowledge on the role of IL-33 in the microenvironment of different tumors. We will highlight which cells produce and which cells are activated by IL-33 in cancer. Furthermore, we will explain how IL-33 modulates the tumor-associated inflammatory microenvironment to restrain or promote tumorigenesis. Finally, we will discuss the issues to be addressed first before potentially targeting the IL-33 pathway for cancer therapy.

## Introduction

Cancer is a heterogeneous disease and represents one of the leading causes of death worldwide. The process generally underlying cancer development was described as the “hallmarks” of cancer (1, 2). These hallmarks include self-sufficient proliferation, insensitivity to antiapoptotic signals, evasion of apoptosis, unlimited replication, maintenance of vascularization, and ability for invasion and metastasis. The so-called tumor microenvironment is also important for tumorigenesis, which supports the hallmarks of cancer. The concept of the tumor microenvironment implies that cancer cells alone are not able to manifest the disease but rather involve resident non-malignant cells or recruit them to participate to tumor development. The interactions between the cancer cells and their supporting stroma result in the formation of tumors and local invasions, metastasis, or vascular niches promoting malignancies (3). The tumor microenvironment consists of immune cells, endothelial cells, pericytes, fibroblasts, and smooth muscle cells (4, 5).

In addition to the occurrence of somatic “driver” mutations in transformed cells, long-term exposure to various environmental stresses, and certain diets may contribute to tumor development (6). Meanwhile, it is also widely accepted that inflammation has an important impact on tumorigenesis (4). The developing tumor itself can promote antitumor immune responses, which is mainly executed by cytotoxic T lymphocytes (CTLs), natural killer cells (NK), but also T helper 1 cells. However, local mechanisms of immune suppression specific to the tumor may blunt these tumor-infiltrating immune effectors (7–9). In addition, inflammatory stimuli may also exert a pro-tumorigenic action. Indeed, about 15% of malignancies are associated with microbes and a subsequent state of chronic inflammation (10, 11). *Helicobacter pylori*, human papillomavirus, hepatitis virus B and C, as well as cytomegalovirus and Epstein–Barr virus have been related to gastric cancer, cervical cancer, hepatocellular carcinoma (HCC), and hematologic malignancies, respectively. Patients suffering from inflammatory bowel diseases (IBDs) have an increased risk of developing colorectal carcinomas (12, 13). Therefore, immune cells and inflammatory mediators are important components of the local environment of tumors (14).

**Figure 1:**
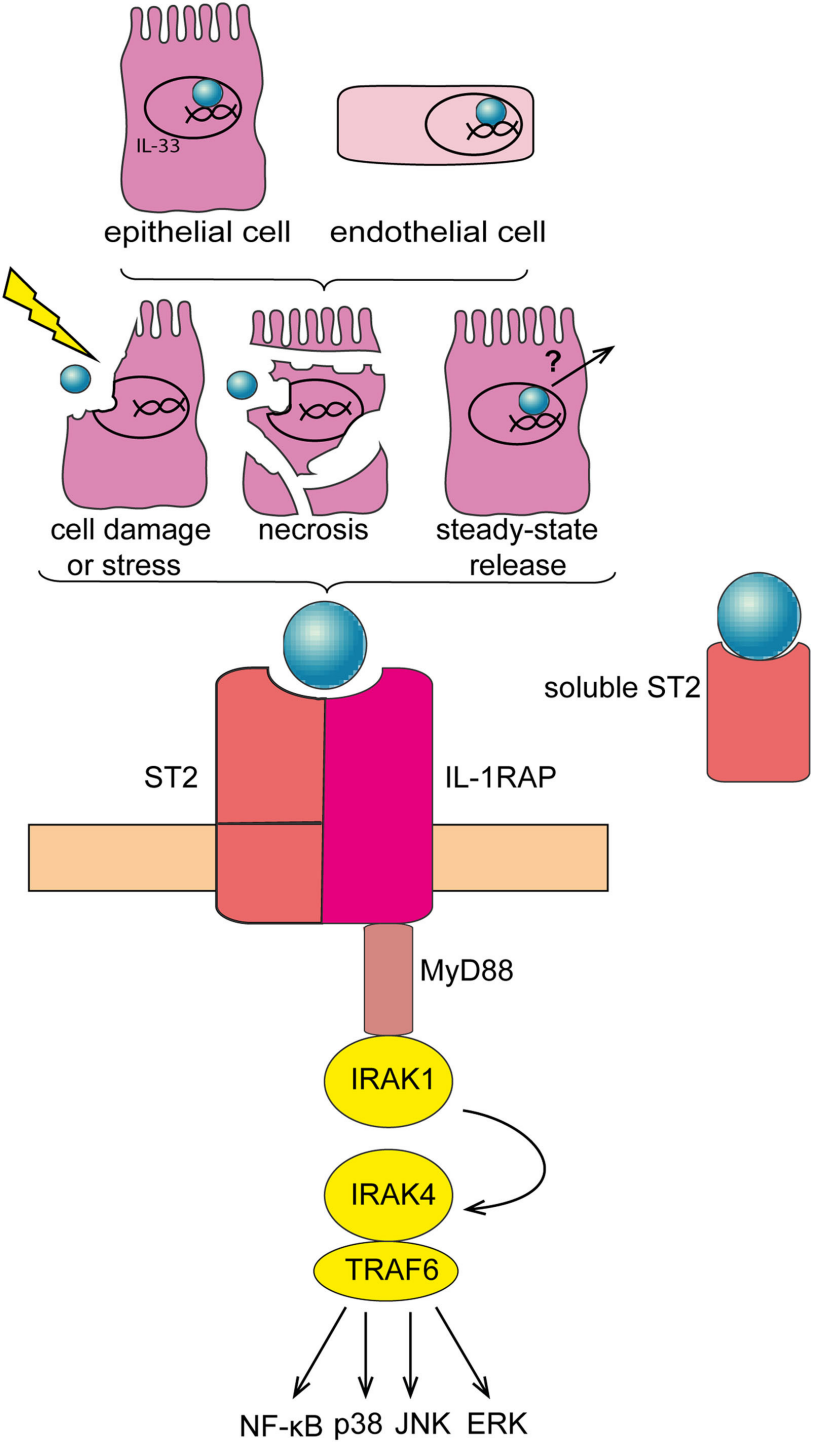
**Interleukin-33 (IL-33)/ST2 signaling pathway**. IL-33 is mainly expressed in the nucleus of epithelial and endothelial cells. IL-33 is released subsequent to cell damage, stress, or necrosis. Alternatively, IL-33 may be released under steady-state conditions—at low levels—*via* a so far unknown mechanism. Soluble IL-33 binds to its receptor, a heterodimeric complex comprising IL-1RL1/ST2 (ST2) and IL-1 receptor accessory protein (IL1RAP), thereby mobilizing downstream signaling molecules including MyD88 and TRAF-6, and eventually activating NF-κB, p38, JNK, and ERK pathways. Soluble ST2 acts as a decoy receptor for IL-33 to negatively regulate IL-33/ST2 signaling.

Cytokines are central mediators of the interaction between cells in the inflammatory tumor microenvironment (15). Interleukin-33 (IL-33) is a member of the IL-1 family of cytokines. In contrast to other IL-1 family members like IL-1β and IL-18, IL-33 is inactivated upon caspase cleavage (16, 17). While full-length IL-33 is already biologically active, its activity is enhanced ~10-fold upon cleavage by neutrophil serine proteases cathepsin G and elastase (18). Full-length IL-33 is mainly expressed by epithelial and endothelial cells, where it is stored and bound to the chromatin in the nucleus (19, 20). IL-33 may exert a dual function, as damage-associated molecular pattern (DAMP) and cytokine (21), or as nuclear factor modulating gene expression (22). Full-length IL-33 acts as “alarmin” if released extracellularly, subsequent to cell stress or damage. Soluble IL-33 binds to its receptor, a heterodimeric complex comprising IL-1RL1/ST2 (which is encoded by *IL1RL1*) and IL-1 receptor accessory protein (which is encoded by *IL1RAP*). IL-33/ST2 signaling is transduced *via* recruitment of MyD88 and IL-1 receptor-associated kinase-4 (IRAK-4), downstream adaptor proteins shared with other IL-1 family members and with most Toll-like receptors (TLRs) (Figure 1). In addition, a soluble form of ST2 (sST2) exists, which is produced by alternative promoter usage, 3′ processing or differential splicing and that may function as a decoy receptor for IL-33 (19, 23–25). IL-33 is primarily known as a driver of type-2 immune responses, triggering the release of Th2 cytokines and thereby promoting allergic reactions (26). Moreover, IL-33 supports tissue repair by coordinating the action of innate lymphoid cells (ILCs) and regulatory T cells (T_regs_) (27–30). Yet, IL-33 may also be involved in pathologic wound repair and fibrosis (31–34). Finally, recent findings have revealed an important contribution of IL-33 to several cancers, where it may exert pro and—less frequently—anti-tumorigenic functions (35).

Here, we review the current knowledge about the role of IL-33 in the microenvironment of different types of tumors. Each cancer type is presented according to the following scheme: (1) short introduction to the specific cancer type; (2) net effect of IL-33/ST2 signaling for tumorigenesis; (3) origin of IL-33- and ST2-expressing cells in the tumor stroma of a particular cancer type; (4) mechanisms of action of IL-33; and (5) therapeutic opportunities. Furthermore, we discuss the major issues which we believe should be investigated in the future to improve our understanding of the role of IL-33 for tumor initiation/progression, toward the development of therapeutic strategies.

## Main Review Text

### IL-33 in Head and Neck Squamous Cancer (HNSCC)

Head and neck squamous cancer represents one of the six leading cancer types worldwide and it arises in the oropharynx, larynx, oral cavity, and hypopharynx. Major risk factors for the development of HNSCC are alcohol consumption and tobacco smoke. However, infection with human papillomavirus (HPV) can also promote tumor development (36).

The tumor microenvironment appears to strongly contribute to HNSCC development. Cancer-associated fibroblasts (CAFs) are the most critical cells in the HNSCC microenvironment, which promote tumor cell proliferation, invasion, and metastatic potential. Therefore, CAFs determine HNSCC tumor aggressiveness. These CAFs release IL-33, which induces epithelial-to-mesenchymal transition of cancer cells, thereby supporting their ability for migration and invasion. Furthermore, IL-33 induces *IL33* gene expression in HNSCC cells *via* a positive feedback mechanism. Increased *IL33* expression in CAFs and in tumor cells is associated with low patient survival (37). In patients with squamous cell carcinoma of the tongue, high expression of IL-33 or ST2 is indicative of worse prognosis. Increased IL-33 levels in these tumors also correlate with microvessels in the stroma (38).

While these data suggest that IL-33 blockade may be beneficial for HNSCC patients, further investigations are required to define downstream key factors dependent on IL-33 for the development of HNSCC. In particular, *in vivo* experiments should be performed to validate these initial data.

### IL-33 in Non-Small-Cell Lung Cancer (NSCLC)

Non-small-cell lung cancer is the most common type of lung cancers, representing more than 85% of all lung cancers. NSCLC has a predicted 5-year survival rate of 15.9%. Although the prognosis for NSCLC has only marginally changed the past years, understanding of the cellular and molecular mechanisms underlying the disease has greatly improved (39).

While many studies have shown a critical role of the IL-33/ST2 signaling in inflammatory lung disease, including respiratory allergy, asthma, and chronic obstructive lung disease (19, 40–42), few have investigated the contribution of this pathway to lung cancer. In NSCLC patients, expression of *IL33* and *IL1RL1* was found to be increased in tumor tissue compared to adjacent non-tumor tissue, and this expression was associated with disease clinical stage (43). However, another study reports downregulation of *IL33* and *IL1RL1* in human lung cancer tissue and cells, compared to normal tissue and cells. Furthermore, the authors found that *IL33* and *IL1RL1* transcript levels were inversely correlated with stages of human lung cancers and overall survival. Accordingly, ST2 protein was expressed in low-metastatic, but not in high-metastatic Lewis lung carcinoma (3LL) cells (44).

Mechanistically, *in vitro* stimulation with IL-33 or overexpression of *IL33* in primary NSCLC cells enhanced their growth and metastasis after transfer into immunodeficient mice. Inversely, shRNA-mediated knockdown of *IL33* in primary NSCLC cells limited their proliferation and invasion capacity *in vivo*. Corresponding results were obtained by overexpressing or downregulating *IL1RL1* in NSCLC cells, thereby showing that the IL-33-driven tumor progression in this model relies on ST2. In addition, engagement of IL-33/ST2 signaling in NSCLC cells was found to increase the membrane expression of glucose transporter 1 (GLUT1) and thereby enhance their glucose uptake and lactate production. Finally, knockdown of *SLC2A1/GLUT1* in NSCLC cells diminished their IL-33-mediated proliferation as well as their metastatic potential, *in vitro* (43).

Yet, IL-33 may also trigger death of ST2-expressing lung tumor cells under conditions mimicking the tumor environment *in vitro*, including upon nutrient depletion or under hypoxic/anoxic conditions. This mechanism may possibly select for more malignant, ST2-negative cancer cells (44).

One can only speculate on the reason for the controversial findings in these two studies. Patient cohorts and cohort size were different in the two investigations. Moreover, no information was provided on the therapeutic status of the analyzed patient samples. Indeed, radiotherapy, alone or in combination with chemotherapy, belongs to the standard treatment of NSCLC (45), which can lead to a rapid increase in the expression of IL-33 protein in tissues (46). Thus, careful evaluation of patient therapeutic history is key for the study of IL-33 signaling in human samples. Finally, while one study describes the use of lung cancer cell lines, primary NSCLC cells were used in the other report, which makes a direct comparison difficult.

Therefore, additional studies are necessary to clarify the contribution of IL-33/ST2 signaling to lung cancer, in particular to NSCLC.

### IL-33 in Breast Cancer

Breast cancer is one of the major causes of cancer-related death among women worldwide (47). Distant metastasis to the lung, bones, liver, and brain are causal for the death of breast cancer patients (48). The immune system has an ambivalent role in breast cancer as it may either promote or prevent tumorigenesis. Tumor-infiltrating leukocytes in particular have a strong effect on breast cancer development. While NK cells and CTLs mediate an anti-tumorigenic response in the microenvironment of breast tumors, T_regs_ and myeloid-derived suppressor cells (MDSCs) act in an immunosuppressive fashion [reviewed in Ref. (49)].

Several studies point toward a pro-tumorigenic role of IL-33/ST2 signaling in breast cancer. IL-33 and ST2 expression are elevated in human breast cancer tissue compared to normal breast tissue (50, 51). In breast cancer patients, serum levels of IL-33 and of its decoy receptor sST2 were enhanced compared to healthy controls. This positively correlated with the expression of vascular endothelial growth factor (VEGF), metalloprotease-11 (MMP-11), or platelet-derived growth factor-C, which are markers of poor prognosis in breast cancer (52).

Compared to wild-type (WT) controls, *Il1rl1*-deficiency led to delayed tumorigenesis in mice tested in the syngeneic 4T1 breast cancer model. Moreover, after orthotopic injection of 4T1 cells, *Il1rl1*-deficient mice showed reduced metastatic potential to the lung and liver. This was caused by decreased tumor cell proliferation in *Il1rl1*-deficient mice. Accordingly, administration of IL-33 in this model enhanced tumor cell proliferation in WT mice, through an indirect effect of IL-33 on tumor cells (53, 54).

In the 4T1 breast cancer model, IL-33 was expressed by CD45-positive leukocytes and tumor cells, while ST2 was mainly expressed on T_regs_ and type-2 ILCs (54). Furthermore, there were fewer MDSCs in tumor lesions of *Il1rl1*-deficient mice, while treatment with exogenous IL-33 promoted the accumulation of these suppressor cells in mammary tumors. MDSCs responded to IL-33 by increasing their production of immunosuppressive TGF-β (54). These MDSCs may be recruited from peripheral lymphoid organs to the tumor lesions, where they trigger the generation of CD4^+^ FoxP3^+^ T_regs_ (55). Indeed, the percentage of CD4^+^ FoxP3^+^ T_regs_, especially the cells expressing ST2 and IL-10, was found to be enhanced within 4T1 mammary tumors.

In addition to these indirect pro-immunosuppressive mechanisms, IL-33 signaling appears to also blunt tumor surveillance in the murine 4T1 breast cancer model. Indeed, NK cells showed increased IFN-γ production in *Il1rl1*^−/−^ mice injected with 4T1 breast cancer cells, which translated into improved antitumor immunity. Along the same line, administration of IL-33 reduced NK cell function *via* decreased expression of FasL or NKG2D and enhanced programed cell death protein 1 (PD-1) levels. IL-33 also altered the ratio of dendritic cell subsets in 4T1 breast tumor lesions (54).

In other models, IL-33 also showed a direct action on malignant breast cells. Stimulation with IL-33 enhanced proliferation as well as colony formation and size of a ST2-expressing breast cancer cell line. This occurred *via* a mechanism involving MAP3K8 phosphorylation upon engagement of IL-33/ST2 signaling (51).

Together, these studies indicate that the IL-33/ST2 pathway promotes breast tumorigenesis both directly, *via* activation of cancer cells, and indirectly, *via* modulation of antitumor immunity. Thus, the IL-33/ST2 pathway may represent an interesting target for breast cancer therapy.

### IL-33 in Hepatocellular Carcinoma (HCC)

Hepatocellular carcinoma is one of the most prevalent cancers and it represents a prominent cause of mortality. Individuals with advanced fibrosis, cirrhosis, and hepatitis B are predisposed to HCC development. Patients with chronic hepatitis B and C infections are most commonly affected. The pathogenesis of HCC is a multistep and complex process, wherein angiogenesis plays an important role (56, 57).

Immuno-histochemical analysis revealed an increase in IL-33-positive tissue in HCC compared to normal liver tissue. In addition, IL-33 serum levels were higher in pre- and post-operative serum samples from HCC patients compared with normal healthy controls (58). However, a different research group found no difference in IL-33 serum levels in HCC compared to liver cirrhosis patients and healthy controls, although levels of sST2 were significantly elevated in their liver cirrhosis and HCC patient groups compared to healthy controls. In HCC patients, serum sST2 levels correlated with overall survival (59).

Yet another study reports reduced levels of IL-33 protein in HCC compared to normal liver, hepatitis, and cirrhosis tissues. Moreover, while IL-33 was observed both in the nucleus and, to a lesser extent, in the cytoplasm of hepatocytes in normal liver tissue, HCC showed only cytoplasmic IL-33 staining. IL-33 expression was negatively associated with HCC histological grade, but not with other parameters including lymph node metastasis. Together, this suggests that cytoplasmic/non-nuclear IL-33 might play a role in HCC development (60).

Although the mechanisms by which IL-33/ST2 signaling may contribute to HCC are still elusive, tumor-infiltrating, IL-33-producing effector-memory CD8^+^ T cells have been observed in resected HCC tissue, which are associated with prolonged patient survival (61).

### IL-33 in Cholangiocarcinoma (CCA)

Cholangiocarcinoma is a malignant neoplasm of the biliary-duct system that represents the second most common primary hepatic malignancy (62). Parasite infections, primary sclerosing cholangitis, biliary-duct cysts, hepatolithiasis, and toxins are known risk factors of this type of cancer (62). Moreover, CCA is also associated with chronic inflammation.

Interleukin-33 has a promoting role for CCA. In a murine model of CCA based on biliary epithelium transduction of constitutively active AKT and Yes-associated protein (YAP) together with bile duct ligation, administration of IL-33 increased biliary tumorigenesis (63). Mechanistically, ST2 protein was expressed in cancer cells and in CAFs, and IL-33 enhanced tumorigenesis through an increase of IL-6 expression in tumor tissue. Moreover, IL-6 was able to substitute for IL-33 in this model (63). IL-6 triggers survival and mitogenic signals in CCA cells, in an autocrine or paracrine fashion (64, 65). Alternatively, administration of IL-33 to mice was found to enhance cholangiocyte proliferation, *via* a mechanism involving an increase in the number of IL-13-producing type-2 ILCs. Activation of this IL-33/ILC2/IL-13 axis in animals with constitutive activation of AKT and YAP in bile ducts induced CCA with liver metastases (66).

Additional *in vivo* experiments for instance using blocking antibodies are necessary to evaluate the relevance of the IL-33/IL-6 or IL-33/IL-13 axis as therapeutic targets in CCA.

### IL-33 in Gastric Cancer

Gastric cancer represents the fourth most common type of cancer and is the second leading cause of cancer-mediated death in the world (67). The 5-year overall survival of gastric cancer patients is only around 20% (68). Major risks for the development of gastric cancer are dietary factors, *H. pylori* infections, gastro-esophageal reflux disease, and obesity [reviewed in Ref. (68)]. Inflammation is an important contributor to gastric tumorigenesis, which is exemplified by the well-studied pro-tumorigenic role of *H. pylori* for this cancer. Indeed, bacterial virulence factors of *H. pylori* and pathogen interaction with the host immune system result in a chronic inflammation that promotes tumorigenesis (69, 70).

Interleukin-33 appears to exert a pro-tumorigenic function in gastric cancer. Human gastric cancer cell lines stimulated with IL-33 showed a dose-dependent increase in cancer cell invasion and migration. These effects were abrogated by knocking-down *IL1RL1*. Engagement of IL-33/ST2 signaling in these gastric cancer cell lines activated ERK1/2 (71), a pathway known to be important for tumor invasion and metastasis (72). Moreover, IL-33 also triggered gastric cancer cells to secrete IL-6 and MMP-3 (71), factors with established pro-tumorigenic properties (73, 74).

Still, additional studies on human gastric cancer tissue and *in vivo* experiments in mice are required to further assess the function of IL-33 for gastric cancer development and progression.

### IL-33 in Colorectal Cancer (CRC)

Colorectal cancer represents one of the most common human cancer types, that is fatal in a considerable number of cases (75). CRC development is a multistep process that follows the adenoma-carcinoma sequence as a consequence of the accumulation of alterations in key oncogenes and tumor suppressor genes involved in the Wnt/β-catenin-, KRAS-, MYC-, MAPK-, TGF-β/bone morphogenetic protein-signaling pathways—among others—and TP53 (76–78). Chronic inflammation strongly promotes intestinal tumorigenesis (4), which is also illustrated by the fact that patients suffering from IBD have a higher risk to develop CRC (79, 80). In addition, several cytokines and inflammatory mediators have been shown to be associated with or to promote intestinal tumorigenesis (81).

While most studies have shown a pro-tumorigenic role of IL-33/ST2 signaling in CRC, recent data suggesting a protective role have also been published. We and others found increased levels of *IL33* and *IL1RL1* expression, both on the transcript and protein level, in adenoma and low grade adenocarcinoma of CRC patients, compared to adjacent normal tissue and high grade tumors (82, 83). Similarly, *IL33* transcript levels were found to be higher in stage I–III CRC compared to adjacent normal colonic tissue. However, *IL33* expression was lower in stage IV CRC than in stage I–III intestinal tumors (84). In contrast, another study reports progressively increasing levels of *IL33* and *IL1RL1* transcript from low to high grade and stage of human CRC (85).

In animal models, *Il33* and *Il1rl1* levels are elevated in adenomatous polyps of *Apc^Min/+^* mice. In this model of CRC, *Il33* deficiency is associated with decreased growth of intestinal polyps (86). Similarly, in the azoxymethane (AOM)/dextran sodium sulfate (DSS) model of colitis-associated CRC (CAC), we found that IL-33/ST2 signaling promotes tumorigenesis. Indeed, *Il1rl1*^−/−^ mice treated with AOM/DSS showed delayed CAC development, fewer/smaller tumors and lesions of lower grade, compared to WT controls. Experiments using bone marrow (BM) chimeric mice indicated that IL-33/ST2 signaling was engaged on both the radio-resistant and the radio-sensitive compartment (83). In a different model of CRC, syngeneic MC-38 colon carcinoma cells injected into the cecum of recipient mice showed enhanced growth and liver metastasis when overexpressing IL-33 (84).

Stimulation of human primary CRC cells with exogenous IL-33 resulted in a dose-dependent increase in their invasive potential. In addition, IL-33 overexpression in these CRC cells resulted in an enhanced invasion, a mechanism dependent on signaling through ST2. These *in vitro* findings were confirmed by challenging nude mice with metastatic SW620 cells. Overexpressing *IL33* in SW620 cells injected subcutaneously enhanced their growth, metastasis, and reduced the survival of recipient nude mice, while downregulating *IL33* had an opposite effect (85).

In human and murine CRC tissue, IL-33 is predominantly expressed by epithelial cells but also in endothelial cells and myofibroblasts. ST2 is mainly expressed on transformed epithelial cells, endothelial cells, myofibroblasts, and infiltrating immune cells (82, 83, 86, 87).

Mechanistically, IL-33 enhances the recruitment of CD11b^+^F4/80^+^ macrophages and CD11b^+^Gr-1^+^ MDSCs to CRC tumors. IL-33 also stimulates the secretion of S100A8/9 (a DAMP) and VEGF from these cell populations to support tumor angiogenesis and metastasis (84).

Several studies have reported an accumulation of mast cells in human and mouse intestinal adenomas, which promote polyposis in *Apc* mutant mouse models *via* stimulation of angiogenesis, modulation of T_reg_ function, and mobilization of MDSCs within the tumor microenvironment (88–90). Along these lines, *Il33*-deficient *Apc^Min/+^* mice showed reduced accumulation of ST2-expressing mast cells in their polyps, compared to *Apc^Min/+^* mice expressing *Il33*. In the same model, abrogation of IL-33/ST2 signaling was associated with reduced expression of proteases and cytokines known to promote polyposis (86).

Stimulation of primary human CRC cells with IL-33 resulted in an increased secretion of IL-6, CXCR4, MMP-2, and MMP-9 (85), which have all been involved in CRC metastasis (91–95). *Apc^Min/+^* mice deficient in *Il33* show reduced transcript levels of *Il4* and *Il6*, which drive CRC development (81, 86). In addition, engagement of IL-33/ST2 signaling on radio-resistant cells decreases the integrity of the intestinal barrier and enables translocation of microbial products to the circulation. This was associated with enhanced systemic levels of pro-tumorigenic IL-6. Furthermore, hematopoietic cells isolated from the vicinity of CAC lesions and stimulated with exogenous IL-33 showed upregulated *Il6* transcript levels. Therefore, the IL-33/ST2 axis contributes to colorectal tumorigenesis partly *via* increased production of IL-6 (83).

Epidermal growth factor (EGF) appears to regulate *Il33* expression in intestinal epithelial cells. In tumors of AOM/DSS-challenged mice treated with gefitinib, an inhibitor of epidermal growth factor receptor, *Il33* transcript levels were decreased, compared to untreated controls. This correlated with fewer and smaller tumors in this model. EGF stimulation of an intestinal epithelial cell line increased its expression of ST2 and intracellular IL-33 (87). This suggests a link between EGF and the IL-33/ST2 signaling axis and may provide a target for the treatment of CRC, although this requires further investigation.

In contrast to the above-presented findings, two recent studies reported an anti-tumorigenic role of IL-33/ST2 signaling for intestinal tumorigenesis. In a first report, expression of transmembrane ST2 was found to be lower in human CRC compared to adjacent, non-tumor tissue, and it progressively decreased during tumor progression from Stage I to Stage IV CRC. While shRNA-mediated knockdown of *Il1rl1* did not alter the proliferation or migration of CT26 CRC cells *in vitro*, these cells developed larger tumors when injected into the flanks of immunocompetent recipient mice. This correlated with a reduction of macrophage recruitment to the tumor tissue, likely due to diminished CCL2 production by tumor cells with abrogated IL-33/ST2 signaling (96). In a second report, IL-33 was shown to promote IgA production, thereby preventing microbial dysbiosis and IL-1α-dependent inflammation in the intestine. Upon AOM/DSS treatment of *Il33*-deficient mice, this translated in augmented secretion of inflammatory cytokines as well as tumors of increased number, size, and grade, compared to WT mice (97). However, it is currently not known whether IL-33 fulfills a similar function in the human intestine, where manipulation of the microbiota may represent a therapeutic strategy for the treatment of CRC, independently of IL-33 (81).

Taken together, the ambivalent role of IL-33/ST2 signaling for CRC tumorigenesis in mouse models makes it difficult to assess this pathway as a possible target for CRC therapy in humans. It seems important to compare the different murine models presented above side-by-side. Moreover, additional experiments should be performed that involve blockade of the IL-33/ST2 pathway at different stages of CRC progression. Also, chemotherapy may differently affect ST2 and IL-33 levels in CRC patients (96), which should be considered in the design of future human studies on these molecules.

### IL-33 in Myeloproliferative Neoplasms (MPNs)

Myeloproliferative neoplasms constitute a group of chronic hematologic malignancies which are characterized by an abnormal proliferation of myeloid cells (98). They are classified as BCR-ABL1-positive MPN, for chronic myeloid leukemia (CML), or BCR-ABL1-negative MPNs, which mainly comprise polycythemia vera, essential thrombocythemia, and primary myelofibrosis (98, 99). Most cases of BCR-ABL-negative MPNs present a mutation in a molecule involved in JAK/STAT signaling (100). As a consequence of these diverse genetic aberrations, cytokine signaling is dysregulated in both classes of MPNs (98).

Interleukin-33/ST2 signaling supports the development of both BCR-ABL1-positive and -negative MPNs. Increased levels of nuclear IL-33 protein are present in trephine biopsies of BCR-ABL1-negative MPN patients, and high amounts of circulating soluble ST2 were detected in the plasma from CML patients, compared to controls. Furthermore, ST2 is upregulated on the surface of CD34^+^ hematopoietic stem/progenitor cells (HSPCs) of BCR-ABL1-positive and -negative MPN patients, compared to healthy donors (101, 102). Addition of exogenous IL-33 increased the colony forming potential of CD34^+^ HSPCs of MPN patients (102).

In mouse models, we found that IL-33 supports MPN-like disease and myeloproliferation in mice deficient in *Inppd5* or in irradiated recipients transfused with BM cells transgenic for human *JAK2*^V617F^, one of the most common driver mutations in MPN patients (102). CD34^+^ HSPCs from CML patients engrafted better in immunodeficient mice after pre-treatment with IL-33, and BCR-ABL-expressing BM cells showed reduced ability to induce CML-like disease when transplanted into *Il33*-deficient versus *Il33*-competent recipients (101).

Interleukin-33 is expressed in radio-resistant cells, including endothelial cells, in the BM. ST2 expression in the BM is mainly restricted to endothelial, mesenchymal, and early myeloid cells. Importantly, ST2 was not detected on murine HSPCs. The IL-33/ST2 pathway in the BM can be activated both in stromal/non-hematopoietic and in hematopoietic cells. Engagement of ST2 on these cells leads to secretion of cytokines known to promote the development and proliferation of myeloid cells, including IL-6, GM-CSF, G-CSF, and IL-3 (102). Since human CD34^+^ HSPCs express ST2, they are able to directly respond to IL-33 stimulation *in vitro*, which induces cytokine production and subsequent cell proliferation (101, 102). These cytokines, in turn, activate the STAT5 pathway and can confer resistance to imatinib mesylate, a specific kinase inhibitor of the BCR-ABL1 fusion protein (101).

While these data suggest a potential therapeutic benefit in blocking IL-33/ST2 signaling in MPNs, additional experiments should first assess the role of this pathway for disease progression and drug resistance in MPN patients.

### IL-33 As Immune Adjuvant for Vaccine Therapy

Except for a few particular examples presented above, IL-33 has mainly a pro-tumorigenic function for cancer development (96, 103). However, IL-33 may also function as an immune adjuvant for antitumor immune response. Indeed, IL-33 has been shown to promote potent cancer-specific effector and memory T cell immunity when used as an adjuvant for DNA vaccination in mice (104). This is in line with findings indicating that IL-33 increases the numbers of CD8^+^ T cells and NK cell-producing IFN-γ in transgenic B16 tumors overexpressing IL-33, thereby mediating a microenvironment favoring tumor rejection. However, IL-33 also promoted the accumulation of immunosuppressive ST2-expressing T_regs_ in this model (103).

Interestingly, aluminum-based adjuvants (alum) induce cell necrosis that leads to extracellular release of IL-33 and the subsequent induction of several IL-33-dependent inflammatory cytokines. In addition, IL-33 injected together with the NP-CGG model antigen increased NP-specific IgM titers in the primary response and T cell-dependent NP-specific IgG1 titers after antigen boost. This indicates that IL-33 can induce robust antigen-specific antibody responses (105). IL-33 was also reported to reduce the accumulation of MDSCs in the spleen and tumor microenvironment, and to decrease the immunosuppressive activity of these cells, which limited tumor growth (106).

Together, these findings indicate the possible use of IL-33 as an immune adjuvant. Yet, there may be some challenges in applying IL-33 for immunomodulation or vaccination, as high levels of IL-33 may lead to lethal inflammatory disease in mice (107, 108). Therefore, it might be difficult to find the right dose of IL-33 to support the desired antitumor-specific immune response while avoiding exacerbated inflammation. This may restrict the possibility of using IL-33 in a vaccine setting.

### IL-33 As Tumor Biomarker or Therapeutic Target

As presented above, IL-33 contributes to the modulation of the tumor environment by promoting the recruitment of pro-tumorigenic immune cells or the secretion of tumorigenic cytokines. However, IL-33 can be detected not only in the tumor environment, but also in the serum of cancer patients. For instance, IL-33 levels are increased in the serum of lung and gastric cancer patients and correlate with disease stage, suggesting that IL-33 may be a negative prognostic marker for these types of cancer (109, 110). Moreover, expression levels of *IL33* and *ST2* correlate with tumor grade and inferior survival of glioblastoma patients (111). However, IL-33 expression indicates favorable prognosis in patients with malignant salivary gland tumors (112). In addition, loss of IL33 expression may also promote tumor escape in patients with metastatic prostate carcinomas or kidney renal clear cell carcinomas, *via* down-modulation of genes involved in antigen processing and of major histocompatibility complex (MHC-I/HLA)-genes (113). Therefore, IL-33 and ST2 differently contribute to tumorigenesis depending on the nature of the malignant tissue.

For breast cancer, IL-33 and sST2 may also serve as non-invasive diagnostic marker, as these two proteins are upregulated in the serum of patients [(52) and see also the paragraph on breast cancer above]. However, IL-33 is upregulated in different types of inflammatory diseases (114–116) and cancers (111, 112), and this lack of specificity may prevent its use as a biomarker in the daily clinic diagnostic. Furthermore, while high levels of IL-33 in absence of common markers of inflammation may indicate the presence of cancer, it would not indicate the identity of the cancerous tissue, and more specific markers would be additionally required for diagnostics. Another level of complexity comes from the observation that the IL-33/ST2 pathway appears to be differently regulated during the progression of distinct types of cancers, as mentioned just above.

While cytokine blockade is currently applied for the treatment of inflammatory disorders, including for instance the use of antitumor necrosis factor-α monoclonal antibodies to treat ulcerative colitis and Crohn’s disease, it remains to be investigated whether a blockade of the IL-33/ST2 pathway may represent a valid approach for the therapy of established IL-33-dependent tumors. Experimental evidences are currently lacking that clearly indicate the suitability of such an approach. Alternatively, IL-33 blockade might be applied in combination with other (immuno)therapies. As IL-33 mainly acts as an amplifier of inflammation (117), targeting IL-33 may suppress the pro-tumorigenic inflammation in the tumor stroma, therefore improving the treatment with conventional therapies. For instance, combination of imatinib and IL-33 blockade in CML may allow elimination of cytokine-dependent malignant stem cells (118) or prevent the emergence of drug resistance (101). Along these lines, combined administration of IL-33 and PD-1 blockade was recently shown to improve the survival of mice suffering from acute myeloid leukemia (119).

## Conclusion and Discussion

**Figure 2:**
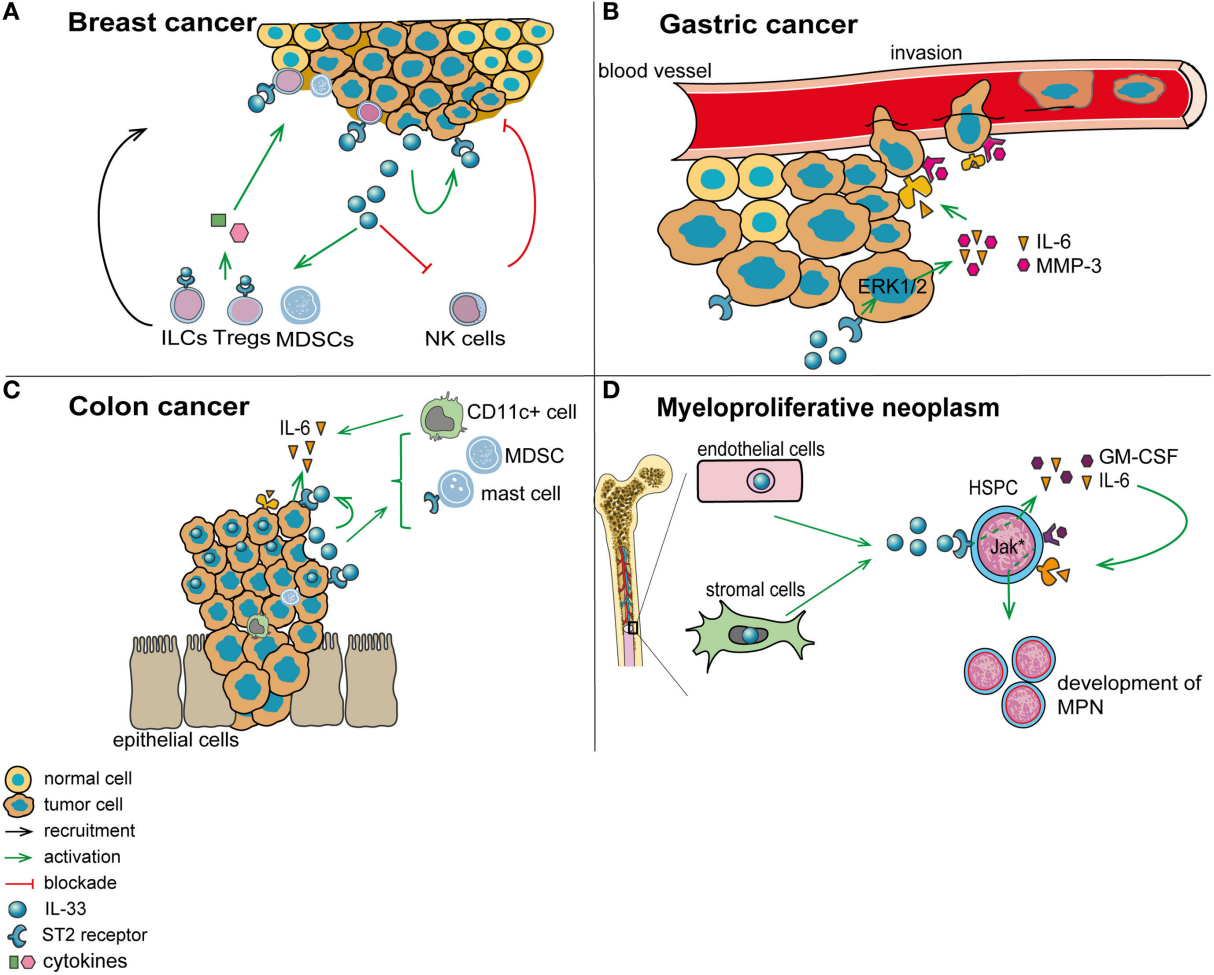
**Role of interleukin-33 (IL-33) in the tumor microenvironment**. **(A)** In breast cancer (mouse 4T1 breast cancer model), IL-33 is released by tumor cells and acts in an autocrine/paracrine manner. In addition, IL-33 promotes the recruitment of immunosuppressive cells and inhibits the function of antitumor cytotoxic natural killer cells (NK). **(B)** In gastric cancer, IL-33 may promote vessel invasion of tumor cells by stimulating the secretion of IL-6 and MMP-3 through activation of the ERK pathway. **(C)** In colorectal cancer, IL-33 supports the recruitment to the tumor environment of pro-tumorigenic immune cells, including mast cells and myeloid-derived suppressor cells (MDSCs). In addition, IL-33 promotes the secretion of pro-tumorigenic IL-6 by leukocytes in the tumor vicinity, which are possibly CD11c^+^ dendritic cells. **(D)** In patients with myeloproliferative neoplasms, IL-33 is released by bone marrow stromal and endothelial cells, and it engages ST2 on CD34^+^ hematopoietic stem/progenitor cells, thereby promoting their secretion of IL-6 and GM-CSF. These cytokines, in turn, stimulate in an auto/paracrine manner the uncontrolled proliferation of the malignant clone (due to its defect in JAK/STAT signaling).

The tumor microenvironment represents a critical incubator for tumor development, local invasion, and metastasis (3). This environment can be modulated by different factors including pro- or anti-tumorigenic cytokines (15). The alarmin IL-33, an amplifier of innate immune response (117), has been shown to contribute to different types of inflammatory diseases and more recently to modulate tumorigenesis (Figure 2) (35, 42). Several investigations using patient-derived material, *in vitro* approaches, or *in vivo* models have uncovered a differential role of the IL-33/ST2 pathway in the tumor microenvironment, for tumor initiation, development, and resistance to therapy (Table 1). While IL-33 has generally a pro-tumorigenic function in various cancers, for some cancer types the findings generated so far are controversial. We discuss below possible reasons for these discrepancies and how they may be possibly addressed or resolved.

**Table 1:** **Interleukin-33 (IL-33) and ST2 levels and contribution in different cancers**.

		Net effect of						
		IL-33			ST2			
Type of cancer		Expression	Tumor progression	Metastasis	Expression	Tumor progression	Metastasis	Major references
Head and neck squamous cancer	Human	+	+					(37, 38)
Non-small lung cancer	Human	+	+	+	+	+	+	(43)
		−	−		−	−		(44)
Breast cancer	Human	+			+			(50, 51)
	Mouse		+			+	+	(53, 54)
Hepatocellular carcinoma	Human	+	+		+			(58, 59)
Cholangiocarcinoma	Mouse		+	+	+			(63, 66)
Gastric cancer	Human		+	+		+	+	(71)
Colorectal cancer	Human	+	+	+	+	+		(82, 83, 85)
		−			−			(96)
	Mouse	+	+	+	+	+		(83, 84, 86)
			−			−		(96, 97)
Myeloproliferative neoplasms	Human	+	+		+			(101, 102)
	Mouse		+			+		(102)

*+, promoting effect; −, suppressing effect*.

### Different IL-33- and ST2-Expressing Cell Types in the Tumor Environment

Several of the expression studies presented above analyzed whole tumor tissues, without distinction of the different IL-33- and ST2-expressing cell types in the tumor microenvironment. However, not only transformed cells, but also infiltrating immune cells, endothelial cells, or myofibroblasts may express IL-33 or ST2 in the tumor stroma (54, 83, 86, 102, 120). Therefore, the presence or contribution of these non-cancerous cells should be separately evaluated for future correlative or functional studies.

The relevance of IL-33- and ST2-expressing non-cancerous cells to tumorigenesis may also explain the contradictory outcomes from distinct animal models. Indeed, xenograft studies are not suitable to address the contribution of stromal cell-specific IL-33/ST2 signaling to tumorigenesis. While there is a 55% homology (19) and a 66% identity at the amino-acid level between human and murine IL-33 and ST2, respectively, it is not known whether these molecules can cross-react between the two species. Moreover, immune cells can engage ST2 upon IL-33 binding, whose contribution cannot be assessed using immunocompromised animals as recipients of xenografts. Finally, for certain types of cancers, heterotopic models may not properly address the role of the physiologic tumor microenvironment. This holds true in particular for CRC, since IL-33 can control dysbiosis in the intestine, which in turn impacts on colon tumorigenesis (97)—a parameter likely omitted by studies involving subcutaneous application of CRC cells. Therefore, these limitations shall be taken into account, especially when evaluating the clinical relevance of the different findings.

### Spatiotemporal Contribution of IL-33 and ST2 to Tumorigenesis

Interleukin-33 may differently contribute to tumorigenesis in dependence on where it is available. As presented above, local IL-33 in the tumor microenvironment can directly trigger cancer or stromal cells. However, IL-33, in particular when given exogenously, may drain to the lymph nodes, where it can promote antitumor responses—as shown in several immune adjuvant vaccine studies (see the above paragraph on IL-33 as immune adjuvant). While this has still to be demonstrated, systemic IL-33 may also possibly keep ST2-expressing immunosuppressive cells at sites distant from (IL-33-expressing) tumors, thereby “distracting” them from their pro-tumorigenic role. Indeed, *in vivo* IL-33 administration can restrict the accumulation of MDSCs in the tumor microenvironment (106).

Finally, IL-33/ST2 signaling may contribute to different stages of tumorigenesis, including tumor growth or metastasis, and additional genetic tools are needed to systematically dissect the role of this pathway at specific time points of cancer development.

### Regulation of IL-33 Release—Role of IL-33 As a Cytokine

As IL-33 may become upregulated in different types of cells upon malignant transformation, its mere presence in tumors should not always imply engagement of ST2-dependent signaling, which may also explain conflicting findings across studies. IL-33 first needs to be released extracellularly before binding to its receptor. However, the exact mechanisms for the regulation and release of IL-33 are still elusive, and they may vary between distinct experimental settings or models. Indeed, in addition to cell damage/necrosis-dependent release of IL-33, IL-33 may also become actively released. Stress conditions associated with increased extracellular adenosine triphosphate (ATP) (a DAMP) may trigger IL-33 secretion after activation of purinergic receptor P2Y2R signaling in live cells (40, 121). Moreover, alternative splicing-mediated deletion of exon 3 and 4 of *IL33* transcripts confers cytoplasmic localization of IL-33 proteins, which facilitates its extracellular secretion from airway epithelial cells of asthmatic patients. Importantly, this isoform of IL-33 was shown to retain its ability to signal through ST2 (122). Thus, it is conceivable that such IL-33 isoforms are also preferentially produced by tumor cells (or cells in the tumor microenvironment), a hypothesis that remains to be addressed. Detectable amounts of IL-33 can be measured in the serum of healthy patients (123) and naïve mice (102) suggesting that IL-33 is released and circulates even under steady-state conditions (Figure 1). Thus, such basal level of IL-33 may also help amplify the inflammatory milieu in chronic inflammatory disorders, which have been shown to provide favorable conditions for the development of tumors (4). Therefore, cancer studies aiming at evaluating the role of IL-33 as a cytokine should systematically assess the function of ST2 in a particular experimental model.

### Role of IL-33 As a Transcriptional Regulator

In addition to its extracellular role as an alarmin triggering ST2, signaling, IL-33 also has a nuclear function. Indeed, IL-33 has been early reported to be expressed in the nucleus of epithelial barrier tissues and lymphoid organs (20, 124). Nuclear IL-33 binds to the p65 subunit of NF-κB, thereby reducing NF-κB-dependent gene expression (22). A study using fibroblast-like synoviocytes from patients with rheumatoid arthritis indicated that downregulation of IL-33 results in increased NF-κB activity and in the production of pro-inflammatory molecules. These results suggest that nuclear IL-33 transcriptionally represses NF-κB and down-modulates inflammatory responses (125). Importantly, NF-κB signaling is not only central for innate immunity and inflammatory processes; it also plays an important role for cancer initiation and progression (4, 126). Therefore, it may be particularly relevant to address the contribution of intracellular IL-33 to NF-κB signaling in tumorigenesis, which has so far not been investigated. As nuclear IL-33 generally becomes upregulated in response to inflammation (40, 127), this may provide a possible control mechanism *via* a negative feedback loop. However, the physiological relevance of this mechanism should be assessed experimentally, as nuclear IL-33 has only limited potential for binding active NF-κB and thus preventing it from binding to DNA (22).

Therefore, this nuclear function of IL-33 may explain why certain investigations using *St2*-deficient mice may not fully recapitulate the findings from *Il33*^−/−^ animals. While the role of nuclear IL-33 needs to be further addressed, e.g., for possible interaction with cancer-relevant transcription factors additional to NF-κB, modulation of nuclear IL-33 expression likely represents a technical challenge for therapy.

Taken together, IL-33/ST2 signaling appears to mainly have a pro-tumorigenic role in cancer. However, several controversial reports provide caveats to a precipitated development of therapeutic strategies to block this pathway, and additional studies are required to unambiguously assess the contribution of IL-33 and ST2 to different cancer types.

## Author Contributions

PK: conception and writing of the manuscript; MHW: writing of the manuscript.

## Conflict of Interest Statement

The authors declare that the research was conducted in the absence of any commercial or financial relationships that could be construed as a potential conflict of interest.

## Abbreviations

AOM, azoxymethane; BM, bone marrow; DC(s), dendritic cell(s); CAC, colitis-associated (colorectal) cancer; CAF(s), cancer-associated fibroblast(s); CCA, cholangiocarcinoma; cDC(s), conventional dendritic cell(s); CRC, colorectal cancer; CTL(s), cytotoxic T lymphocytes; DSS, dextran sodium sulfate; EGF, epidermal growth factor; EGFR, epidermal growth factor receptor; HCC, hepatocellular carcinoma; HNSCC, head and neck squamous cancer; HSPC(s), hematopoietic stem/progenitor cell(s); IBD, inflammatory bowel disease; IL, interleukin; ILC(s), innate lymphoid cell(s); MDSC(s), myeloid-derived suppressor cell(s); MMP, metalloprotease; NK, natural killer cells; NSCLC, non-small-cell lung cancer; pDC(s), plasmacytoid dendritic cell(s); PDGF, platelet-derived growth factor; sST2, soluble form of ST2; TNF, tumor necrosis factor; T_reg(s)_, regulatory T cell(s); VEGF, vascular endothelial growth factor; WT, wild type.
